# Supplementary material for: A systems-level insight into PHB-driven metabolic adaptation orchestrated by the PHB-binding transcriptional regulator AniA (PhaR)
Source: mSystems. 2025 Sep 22;10(10):e00760-25. doi: 10.1128/msystems.00760-25 (PMC12542648; doi:10.1128/msystems.00760-25)
Supplement: Supplemental figures — Figures S1 to S3. [file msystems.00760-25-s0001.pdf]

Supplemental Figures

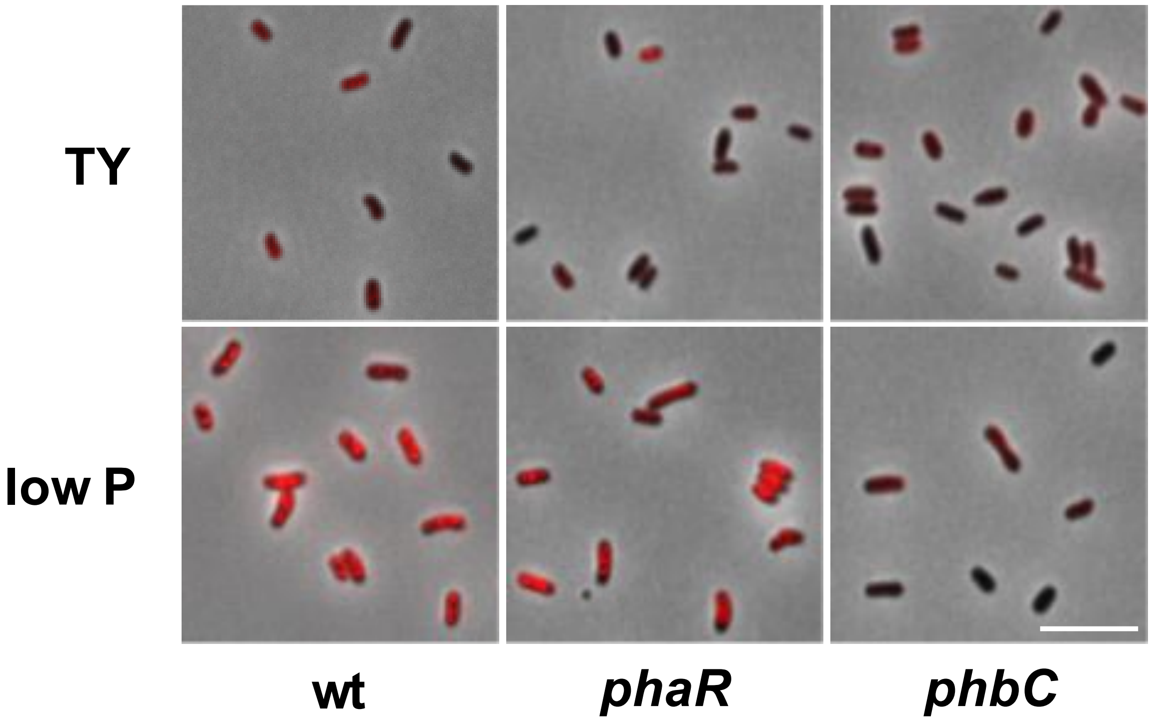

Figure S1. Microscopy images of Nile red-stained cells after 24 hours of growth in TY or low-phosphate (low P) medium. Scale bar: 5 µm.

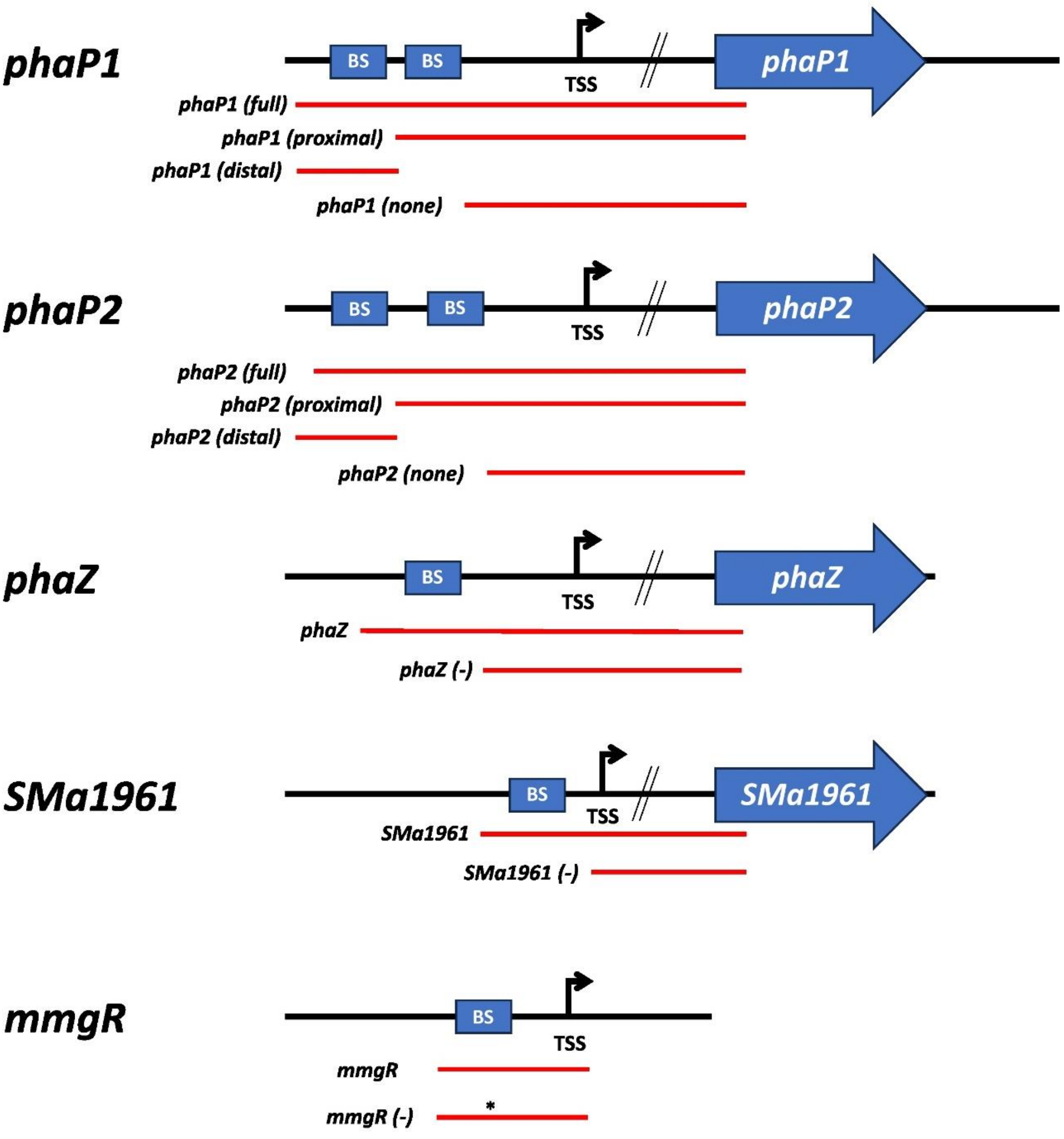

Figure S2. Schematic representation of promoter variants of PHB-related genes used for EMSA. The genomic regions corresponding to each probe are indicated with red lines. **BS**: PhaR-binding site. **TSS**: transcriptional start site. An asterisk in the *mmgR* (–) probe indicates the mutation of the palindromic sequence *TGCACCGCA* to *TGCACCAAA* to generate a mutant *mmgR* promoter probe.

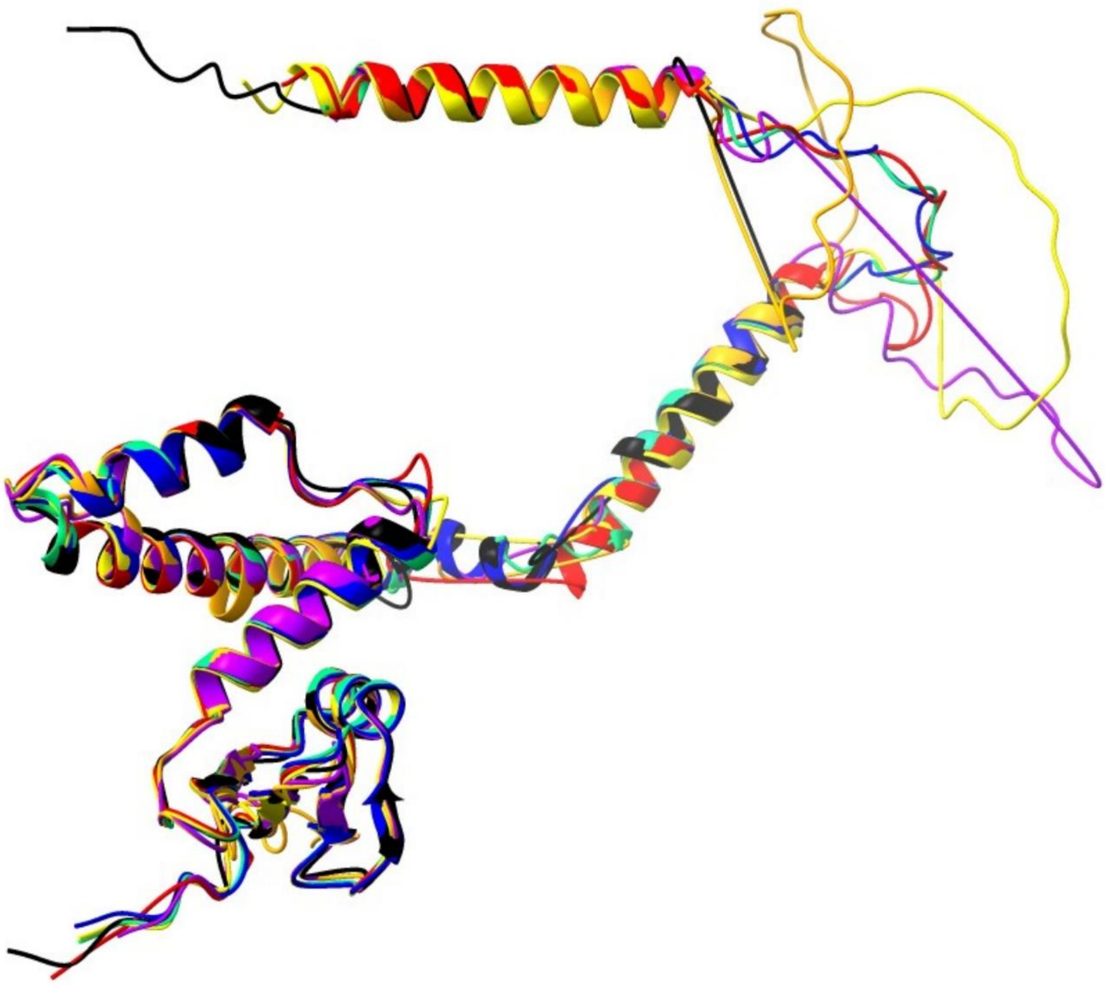

Figure S3. Multiple flexible protein structure alignment of PhaR homologs from *S. meliloti* 2011 (green, AlphaFold accession AF-Q7APA2-F1), *R. etli* CFN42 (blue, AlphaFold accession AF-Q2K325-F1) *M. extorquens* DM4 (red, AlphaFold accession AF-C7CDY8-F1) *C. necator* N-1 (black, AlphaFold accession AF-Q0KBP6-F1) *B. diazoefficiens* USDA110 (yellow, AlphaFold accession AF-Q89XT0-F1) *R. spheroides* DSM158 (purple; AlphaFold accession AF-Q3J0X9-F1) *P. denitrificans* Pd 1222 (orange, AlphaFold accession). Protein structures were downloaded from the AlphaFold database on Dec 27, 2024. The flexible structure alignment was performed with the Kpax software (Ritchie, 2016). The structure of *S. meliloti* PhaR was used as pivot by setting -nopivot, and flexible (-flex) and multiple (-multi) options were enabled. The reported M-score was 0.86168.
