# Supplementary material for: A systems-level insight into PHB-driven metabolic adaptation orchestrated by the PHB-binding transcriptional regulator AniA (PhaR)
Source: mSystems. 2025 Sep 22;10(10):e00760-25. doi: 10.1128/msystems.00760-25 (PMC12542648; doi:10.1128/msystems.00760-25)
Supplement: Supplemental Methods — Quantitative proteomic analyses. [file msystems.00760-25-s0002.pdf]

Quantitative proteomic analyses. A bacterial biomass from 1 ml of cultures that were grown for 48 h in TY and MOPS-buffered low P media was collected by centrifugation at 10,000 × *g* for 3 min at RT. The pellets were resuspended in 150 µl of milliQ water and transferred to a screw-cap 2-ml tube filled with ca. 70 mg of 0.1 mm glass beads. Bacterial cells were lysed by four 60-sec-cycles of homogenization at 6.5 m/s in a FastPrep-24 device (MP Biomedicals). Then, 150 µl of a 2× lysis buffer (40 mM HEPES pH 7.5, 4% Sodium Lauryl Sarcosinate) was added to the suspensions and the samples were further incubated at 90°C for 15 min. The lysates were clarified by centrifugation at 20,000 × *g* for 5 min at RT, and the supernatants were stored at -20°C. The total protein content in the lysates was quantified with the Bicinchoninic Acid method (BCA Protein Assay Kit), and a sample volume containing 50 µg of protein was submitted for proteomic analysis.

Proteins were reduced with 5 mM Tris(2-carboxyethyl) phosphine (Thermo Fischer Scientific) at 90°C for 15 min and alkylated using 10 mM iodoacetamid (Sigma Aldrich) at 20°C for 30 min in the dark. Proteins were precipitated with a 6-fold excess of ice-cold acetone, followed by two methanol washing steps. Dried proteins were reconstituted in 0.2 % SLS and the amount of proteins was determined by the bicinchoninic acid protein assay (Thermo Scientific). For tryptic digestion 50 µg protein was incubated in 0.5% SLS and 1 µg of trypsin (Serva) at 30°C overnight. After digestion, SLS was precipitated by adding a final concentration of 1.5% trifluoroacetic acid (TFA, Thermo Fischer Scientific). Peptides were desalted by using C18 solid phase extraction cartridges (Macherey-Nagel). Cartridges were prepared by adding acetonitrile (ACN), followed by equilibration with 0.1% TFA. Peptides were loaded on equilibrated cartridges, washed with 5% ACN and 0.1% TFA containing buffer and finally eluted with 50% ACN and 0.1% TFA. Peptides were dried and reconstituted in 0.1% trifluoroacetic acid and then analyzed using liquid chromatography-mass spectrometry carried out on a Exploris 480 instrument connected to an Ultimate 3000 RSLC nano and a nanospray flex ion source (all Thermo Scientific). Peptide separation was performed on a reverse phase HPLC column (75 µm x 42 cm) packed in-house with C18 resin (2.4 µm; Dr. Maisch). The following separating gradient was used: 94% solvent A (0.15% formic acid) and 6% solvent B (99.85%

acetonitrile, 0.15% formic acid) to 25% solvent B over 95 minutes at a flow rate of 300 nl/min, and an additional increase of solvent B to 35% for 25 min. MS raw data was acquired in data independent acquisition mode with a method adopted from Bekker-Jensen et al. (Bekker-Jensen et al., 2020). In short, Spray voltage were set to 2.3 kV, funnel RF level at 40, and heated capillary temperature at 275 °C. For DIA experiments full MS resolutions were set to 120.000 at m/z 200 and full MS, AGC (Automatic Gain Control) target was 300% with an IT of 50 ms. Mass range was set to 350–1400. AGC target value for fragment spectra was set at 3000%. 45 windows of 14 Da were used with an overlap of 1 Da. Resolution was set to 15,000 and IT to 22 ms. Stepped HCD collision energy of 25, 27.5, 30 % was used. MS1 data was acquired in profile, MS2 DIA data in centroid mode.

Analysis of DIA data was performed using DIA-NN version 1.8 using a UniProt protein database from *Sinorhizobium meliloti* 1021 (Consortium, 2023; Demichev et al., 2020). Full tryptic digest was allowed with two missed cleavage sites, and oxidized methionines and carbamidomethylated cysteine. Match between runs and remove likely interferences were enabled. The neural network classifier was set to the single-pass mode, and protein inference was based on genes. Quantification strategy was set to any LC (high accuracy). Cross-run normalisation was set to RT-dependent. Library generation was set to smart profiling. DIA-NN outputs were further evaluated using the SafeQuant and script modified to process DIA-NN outputs (Ahrné et al., 2013; Glatter et al., 2012). The SafeQuant script was executed on the “report.tsv” file from DIA-NN analysis to sum precursor intensities to represent protein intensities. The peptide-to-protein assignment was done in SafeQuant with redundant peptide assignment following the Occam´s razor approach. Median protein intensity normalization was performed.

The statistical analysis was performed in Perseus (Tyanova & Cox, 2018) and R (Team, 2014). Proteins with at least two unique peptides identified were considered for further analysis. To ensure robust detection, a protein was retained if at least two unique peptides were detected in at least two out of three biological replicates for at least two out of the four mutant strains.

Missing values for proteins meeting these criteria were imputed using a Gaussian distribution with a standard deviation of 0.3 and a downshift of 1.8 standard deviations, with these parameters set separately for each sample's proteome. ANOVA analyses of proteomes from cells grown in low P or TY medium were performed in Perseus. The results were filtered using a permutation-based false discovery rate (FDR) correction, with a significance threshold for q-values set at 0.05. Z-score data normalization was carried out in Perseus accessing the matrix by rows with grouping set by growth medium. Principal component analysis (PCA) was performed using the Factoextra package for R (Kassambara & Mundt, 2020). Z-scored data was used to run a co-expression network analysis with the hCoCena package for R (Oestreich et al., 2022), following the protocol recently published by the developers (Holsten et al., 2024). Enrichment analysis was performed using a Fisher’s Exact Test to assess the overrepresentation of functional categories among co-expression modules. The analysis was conducted using Perseus, and p-values were corrected for multiple testing using the Benjamini-Hochberg false discovery rate (FDR) method.
